# A Colorimetric Dip Strip Assay for Detection of Low Concentrations of Phosphate in Seawater

**DOI:** 10.3390/s21093125

**Published:** 2021-04-30

**Authors:** Hojat Heidari-Bafroui, Amer Charbaji, Constantine Anagnostopoulos, Mohammad Faghri

**Affiliations:** Microfluidics Laboratory, Department of Mechanical, Industrial and Systems Engineering, University of Rhode Island, Kingston, RI 02881, USA; charbaji@uri.edu (A.C.); anagnostopoulos@uri.edu (C.A.); faghrim@uri.edu (M.F.)

**Keywords:** phosphate detection, paper-based device, molybdenum blue method, colorimetric assay

## Abstract

Nutrient pollution remains one of the greatest threats to water quality and imposes numerous public health and ecological concerns. Phosphate, the most common form of phosphorus, is one of the key nutrients necessary for plant growth. However, phosphate concentration in water should be carefully monitored for environmental protection requirements. Hence, an easy-to-use, field-deployable, and reliable device is needed to measure phosphate concentrations in the field. In this study, an inexpensive dip strip is developed for the detection of low concentrations of phosphate in water and seawater. In this device, ascorbic acid/antimony reagent was dried on blotting paper, which served as the detection zone, and was followed by a wet chemistry protocol using the molybdenum method. Ammonium molybdate and sulfuric acid were separately stored in liquid form to significantly improve the lifetime of the device and enhance the reproducibility of its performance. The device was tested with deionized water and Sargasso Sea seawater. The limits of detection and quantification for the optimized device using a desktop scanner were 0.134 ppm and 0.472 ppm for phosphate in water and 0.438 ppm and 1.961 ppm in seawater, respectively. The use of the portable infrared lightbox previously developed at our lab improved the limits of detection and quantification by a factor of three and were 0.156 ppm and 0.769 ppm for the Sargasso Sea seawater. The device’s shelf life, storage conditions, and limit of detection are superior to what was previously reported for the paper-based phosphate detection devices.

## 1. Introduction

Phosphorus (P) plays a crucial role in the growth of marine life as an essential nutrient for living organisms. However, in an aqueous solution, a fully oxidized form of phosphorus, i.e., phosphate, takes a critical part in the eutrophication process. Phosphates are categorized into three forms: orthophosphates, condensed phosphates, and organic phosphates. In the monitoring of water quality, phosphates are commonly referred to as orthophosphates [1]. Excessive concentrations of phosphate due to the predominant usage of phosphate-based pesticides and fertilizers [2], along with intentional or accidental inappropriate human activities [3,4], are known to be harmful to environmental aquatic systems. The US Environmental Protection Agency (US EPA) has set the desired limit of 0.150 ppm or 150 parts-per-billion (ppb) for the total phosphate concentration in streams flowing into a lake or reservoir [5]. Thus, in order to preserve the quality of water within an acceptable range of phosphate, it is crucial to monitor the phosphate content in these environments. The traditional methods of quantitative water analysis use specialized and expensive equipment with highly qualified operators to analyze samples taken from sites and shipped to laboratories [6]. Therefore, the detection of low phosphate and other nutrient concentrations in the field, rapidly and effectively in complex aqueous matrixes, has become an exciting and active area of research [7,8,9].

A wide range of analytical methods have been developed for phosphate concentration in aquatic samples so far, including electrochemical analysis [10,11], spectrophotometry [12,13], colorimetry [14,15], fluorometry [16,17], and enzymatic biosensors [18,19]. However, the Molybdenum blue-based spectrophotometry method introduced by Murphy and Riley [20] and improved by successive researchers [21,22,23] has been broadly accepted as a standard method for the determination of phosphate in water [1]. In this method, ammonium molybdate in a strong acidic condition reacts with orthophosphate (Equation (1)) to form a Keggin ion [PMo_12_O_40_]^3−^ and then is reduced by ascorbic acid (Equation (2)) to generate the molybdenum blue complex [24]. Potassium antimonyl tartrate, as a source of antimony, has been frequently used for increasing the rate of reaction and eliminating the need for a heating process to form the stable molybdenum blue product [25,26,27].

PO_4_^3−^ + 12MoO_4_^2−^ + 27H^+^ ⟶ H_3_PO_4_(MoO_3_)_12_ + 12H_2_O (1)

H_3_PMo(VI)_12_O_40_ + Ascorbic acid ⟶ [H_4_PMo(VI)_8_Mo(V)_4_O_40_]^3−^ (2)

One recent method for conducting inexpensive analysis of nutrients in water, which is field deployable, disposable, and easy-to-use by an unskilled operator, utilizes paper-based devices and the colorimetric method [28]. Thus far, only three colorimetric paper-based assays have been proposed for the detection of phosphate in water. Jayawardane et al. [29] was the first research group that developed a 3D paper-based device in which an interleaving Teflon sheet was used to separate two reagents deposited on filter paper. They stored the chromogenic reagent on paper, and it consisted of ammonium molybdate and potassium antimony (III) tartrate dissolved in a high concentration of sulfuric acid (6.6 M). Because of the auto-reduction of molybdenum in acidic conditions [24], the lifetime was only a couple of days in ambient conditions and was improved to 122 days when the device was stored in a freezer at <−20 °C. Ribeiro [30] and Racicot et al. [31] tried to stabilize the molybdenum reagent by adding ethylene glycol. Racicot et al. reported an improved shelf life of the device of up to 35 weeks. However, they evaluated the degradation of the molybdenum reagent in the presence of ethylene glycol when dried on paper and stored at <−4 °C. Nevertheless, when running the experiments to measure the limits of detection and quantification for their device, the molybdenum reagent was stored in a pre-filled syringe in liquid form and not dried on paper. Moreover, the way that their device works does not follow the appropriate sequence for the two standard reactions in the molybdenum blue method. In their device, the two reagents mix together and the redox reaction initiates before even applying the phosphate sample, which increases the variability of the color intensity that forms in the detection zone. Finally, Waghwani et al. [32] developed a paper-based assay based on the molybdenum yellow method. However, their device was able to detect concentrations of phosphate ranging from 1 mg/mL to 20 mg/mL (1000 to 20,000 ppm), which means the sensitivity of their device is not even sufficient for the detection of phosphate in wastewater [33,34]. Hence, there is a need for a robust, reliable, portable, and stable device with a long shelf-life to detect low concentrations of phosphate.

In this paper, we report the development of a system that combines wet and dry chemistry in a paper-based device in order to detect low concentrations of phosphate in water and seawater. Molybdate dissolved in deionized (DI) water and sulfuric acid were stored separately to enhance the lifetime of the device. Ascorbic acid and antimony were stored in dried form on a paper strip.

## 2. Materials and Methods

### 2.1. Solution Preparation

A stock solution of phosphate (100 ppm) was freshly created by dissolving 0.0126 g sodium dihydrogen phosphate (Sigma-Aldrich, MO, USA) in 100 mL of ASTM Type 1 deionized water (resistivity > 18 MΩ/cm, LabChem-LC267405). Then, by diluting the stock solution with DI water, a series of phosphate solutions with concentrations 0.1, 0.25, 0.50, 0.75, 1, 2.5, 5, 7.5, 10, and 25 ppm were prepared. In addition, a real seawater sample taken from the Sargasso Sea was filtered through a 0.2 µm filter to remove any organic matter and was then used to evaluate the performance of the device in the presence of the ions usually found in seawater. The Sargasso Sea region is recognized as a region with low nutrient content [35].

### 2.2. Reagents

All chemicals are analytical reagent grade and were obtained from Sigma-Aldrich (Saint Louis, MO, USA). Each glassware and vial was first washed with a phosphate-free detergent and then washed with 1 M hydrochloric acid and rinsed with DI water three times. The development of the molybdenum blue complex and its stability mainly depends on [H^+^], ammonium molybdate, and ascorbic acid concentrations in the final solution. These concentrations have been thoroughly optimized, and the interference studies were reported in [29]. The concentrations in the final solution for the device presented in this study are the same with [29] except for the concentration of [H^+^], i.e., the concentration of sulfuric acid. Molybdenum reagent was prepared by dissolving 1.054 g ammonium hepta-molybdate tetrahydrate in 10 mL DI water and stored in a plastic dropper bottle. This solution is indefinitely stable in room conditions [36,37]. A total of 10 mL of 3.6 M sulfuric acid was created by first slowly adding 2.02 mL of 95% *w*/*w* sulfuric acid to 2.5 mL deionized water and then by adjusting the final volume of solution to 10 mL with DI water. This solution was stored in a glass dropper bottle, and its expiration date is two years, according to the supplier. The reducing reagent was prepared as a solution of 0.5 M ascorbic acid and 6 mM potassium antimony tartrate hydrate in DI water.

### 2.3. Device Preparation

**Figure 1:**
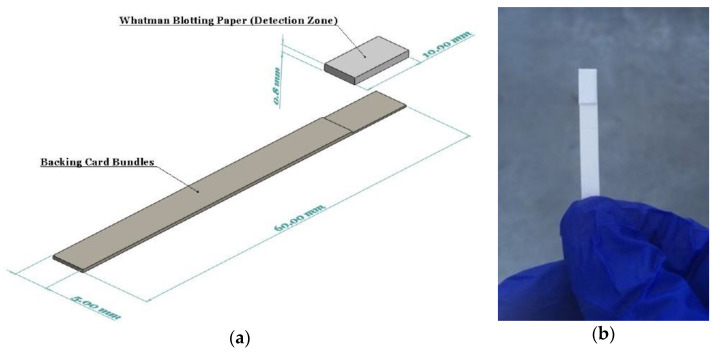
(**a**) A schematic showing the components and dimensions of the dip strip; (**b**) The assembled device with ascorbic acid and antimony dried on the detection zone.

Whatman blotting paper (WHA10547922-Whatman^®^ gel blotting paper, Grade GB003) strips (10 × 100 mm) were fully saturated with the reducing reagent by immersing in the solution for 5 min. The strips were then allowed to air dry in room conditions for two hours. This paper grade was selected since it is made from pure cellulose with high absorbency and without any additives. The dried reducing reagent used on the detection zone was entirely stable on the strips for several months. A pair of backing cards, 60 mm wide by 100 mm long, each ~0.254 mm thick (MIBA-010, DCNovations, CA, USA), were utilized to stick the reducing strips on them so as to improve the strength and allow for easy handling of the strips by the operator. These backing cards are specifically produced for use in lateral flow devices. They have an acrylic pressure-sensitive and non-reactive adhesive that supports the devices built on them, as well as provides minimum interference [38]. Finally, a guillotine cutter was used to cut the dip strips with a width of 5 mm (Figure 1).

### 2.4. Device Operation and Analysis Procedure

**Figure 2:**
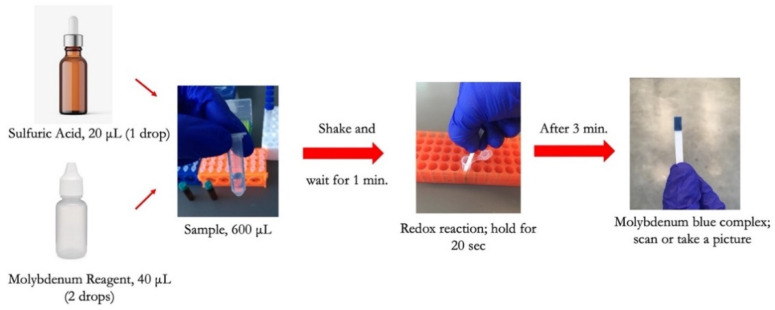
Illustration of phosphate detection procedure using the dip strip.

**Figure 3:**
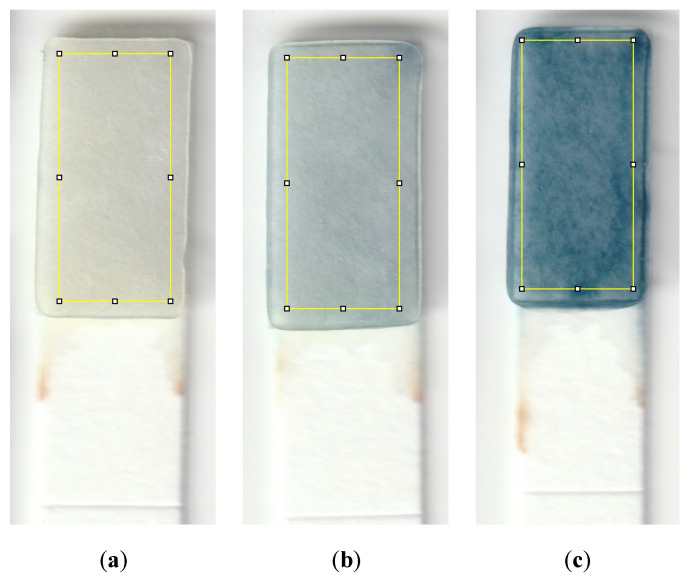
Detection zones showing the color that forms for a sample with a phosphate concentration of (**a**) 0.1 ppm, (**b**) 1 ppm, and (**c**) 10 ppm. The yellow rectangle is the region of interest utilized to quantify the color intensity using ImageJ; this area is 95 by 215 pixels, which is approximately 4 by 9 mm.

To conduct the test, a small sample volume of 600 μL was mixed with 20 μL of sulfuric acid in a micro vial, and the mixture was properly shaken by hand for 5 s. 40 μL of molybdenum reagent was added to the mixture, shaken, and allowed the formation of the Keggin ions for one minute. The strip was then dipped into the solution for 20 s, and then the color was allowed to form for 3 min before capturing an image from the detection zone using a desktop scanner (Canon TS6020) at a resolution of 600 DPI. Figure 2 is a diagram showing the steps needed to detect phosphate using the dip strip. The intensity of the red color was subsequently measured by ImageJ software to compute the limits of detection and quantification. The red color was chosen since the final color formed on the strip is blue, which means the red color is mostly absorbed and shows the largest range of value corresponding to the change in phosphate concentration. Figure 3 shows the evolution of color formation with an increase in phosphate concentration. It also shows the region of interest used to quantify the color intensity using ImageJ.

### 2.5. Portable Infrared Lightbox

**Figure 4:**
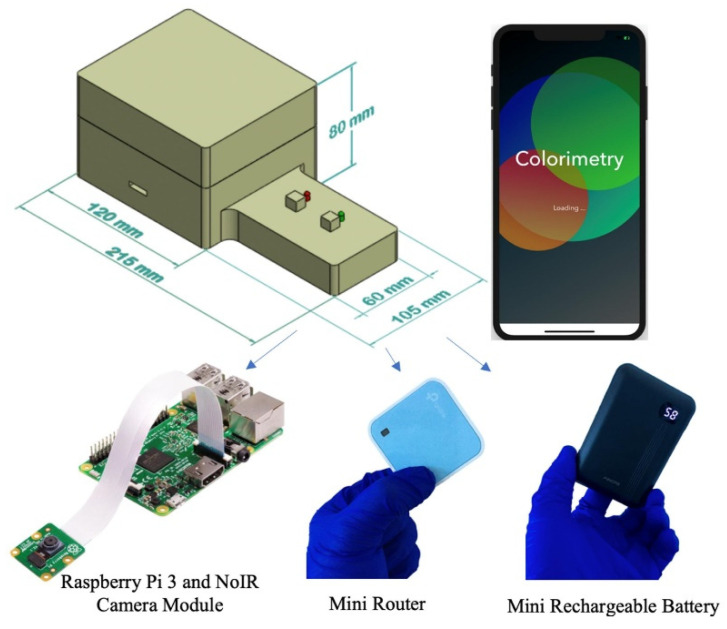
Schematic of the lightbox and its components that is wirelessly controlled by a smartphone to capture images of the detection zones in the infrared zone [39].

An inexpensive and portable lightbox was previously developed to improve the detection limits of paper-based devices for detecting phosphate [39]. This lightbox measures the absorbance of the molybdenum blue reaction in the infrared region since the peak absorbance of this reaction occurs in the infrared zone of the spectrum. The lightbox includes infrared light-emitting diodes, a digital camera without an infrared filter, a Raspberry Pi microcontroller, a mini-router, and a mini rechargeable battery for the onboard power supply. The mini router allows the lightbox to wirelessly interface with a smartphone in order for the user to remotely command the microcontroller and capture images of the detection zones. Figure 4 shows a schematic of the lightbox and its components.

## 3. Results and Discussion

### 3.1. Timing Optimizations for the Device

**Figure 5:**
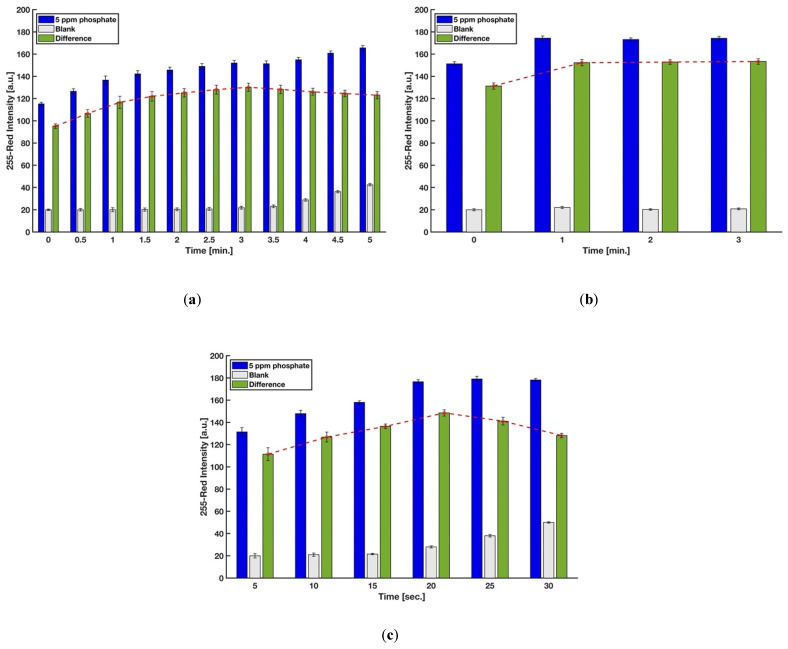
Effect of (**a**) reaction time, (**b**) the time for mixing the molybdenum reagent with the acidic sample, and (**c**) the time for dipping the strip into the mixture solution on color intensity (*n* = 3 and the error bars show the standard deviation).

In this procedure, the timing for scanning, mixing of the molybdenum reagent with the acidic sample, and dipping the strip into the mixture was precisely controlled so as to achieve accurate results. The reaction time for generating the molybdenum blue complex on the detection zone was first studied for phosphate concentrations of 0 ppm (blank) and 5 ppm. Image capture for each device was done every 30 s by the scanner, and the red intensities were recorded. The difference between the red intensities of 5 ppm and 0 ppm was the precise controlling indicator to eliminate the effect of the auto-reduction of molybdenum when there are no phosphate ions in the sample. Figure 5a shows that the difference in color intensity between 0 and 5 ppm kept increasing up to 3 min, and a gradual decrease of color intensity was observed after that. Hence, the reaction time of 3 min was chosen for further experiments. However, the analysis of the results for 2.5, 3, 3.5, and 4 min using a one-way ANOVA method demonstrated that the color intensities are not significantly different (*F*(3,9) = 1.56, *p*-value = 0.27, at the 95% confidence level), which means the test kit is not highly sensitive for a short time interval.

The mixing time when molybdenum reagent was added to the mixture of sulfuric acid and sample and prior to dipping the strip was also evaluated in order to obtain the higher color formation on the detection zone. As indicated in Figure 5b, waiting for at least one minute improved the difference between red intensities of 5 and 0 ppm phosphate solutions. This short time is needed for the heteropoly acids to completely form in the entire solution before the addition of the reducing agent. Lastly, six levels for the holding time, i.e., holding of the test strip into the micro vial filled by the sample, sulfuric acid, and molybdenum reagent, were considered in order to study the effect of this time, which is proportional to the amount of the phosphomolybdic acids (H_3_PMo(VI)_12_O_40_) absorbed by blotting paper. Figure 5c implies that 20 s is the optimum time for having the highest amount of molybdenum blue complex on the detection zone after the test operation.

### 3.2. Optimization of Sulfuric Acid’s Volume

**Figure 6:**
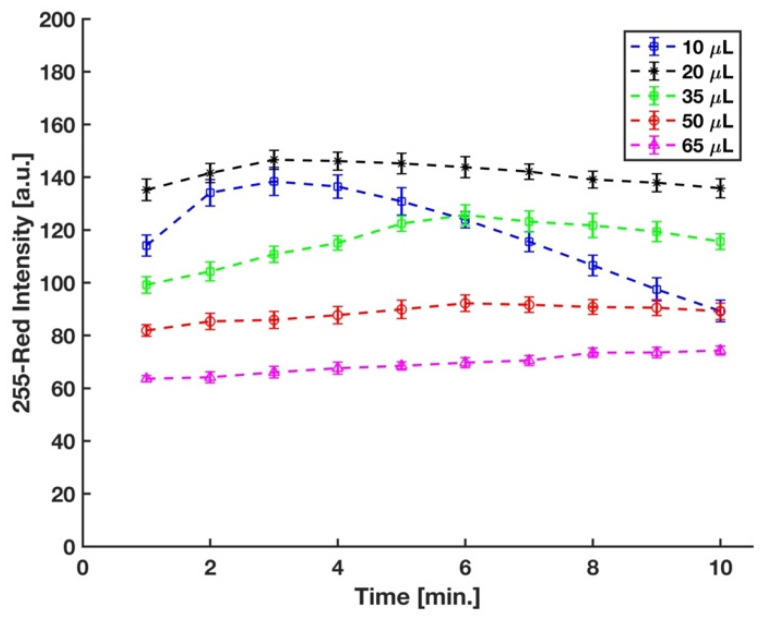
Effect of volume of sulfuric acid in the mixture on color intensity (*n* = 3 and the error bars show the standard deviation).

In the experimental setup, the sulfuric acid concentration was controlled by the volume of acid added to the micro vial. Acid concentration plays a significant role in the formation of the phosphomolybdenum blue, as well as its rate of formation. It has been well-established that a strong acid condition is needed to prevent auto-reduction of molybdenum in the absence of phosphate [24]. Different strong acids, such as sulfuric acid, hydrochloric acid, perchloric acid, and nitric acid, have been used in the molybdenum blue method for phosphate detection [40,41,42,43]. However, sulfuric acid has been mostly preferred in this method since nitric and perchloric acids (oxidizing acids) interfere with the reduction reaction of phosphomolybdenum acid [24,44], and the chloride ions in the hydrochloric acid hinder the development of the molybdenum blue complex [45]. Therefore, for this study, sulfuric acid was used, and the optimum volume of 3.6 M sulfuric acid in the final solution was determined by measuring the difference between the red intensity of complex developed on the detection zone of the device by the 5 ppm and 0 ppm (blank) phosphate samples at five different sulfuric acid volumes. We started with 50 μL sulfuric acid to have the same concentration of the acid in the final solution with Ref. [29]; however, as shown in Figure 6, the maximum sensitivity was reached with 20 μL of sulfuric acid. 

### 3.3. Role of Antimony in Phosphate Detection

The use of antimony (Sb) in the molybdenum blue methodology was introduced by Murphy and Riley in 1962 [20] to greatly accelerate the reduction process by ascorbic acid without a need for prolonged heating steps. They determined that the final solution contains antimony and phosphorus (Sb:P) in a 1:1 atomic ratio. However, it has long been shown that a 2:1 ratio of Sb:P is present in the reduced complex instead of a 1:1 ratio [21,23], while the stoichiometry of the reduced phosphoantimonylmolybdate species and the reasons for the accelerating effect of antimony on the reduction kinetics have not completely been elucidated [24]. It was proposed that two Mo(VI) atoms may be substituted by two Sb(III) atoms in the Keggin structure, and the form of complex is probably [PSb_2_Mo_10_O_40_]^n−^ [21]; however, an electrospray ionization mass spectrometry (ESI-MS) study established that Mo is not replaced by Sb in the Keggin ion, and there is no observation for the presence of [PSb_2_Mo_10_O_40_]^n−^ in the final complex [46,47].

**Figure 7:**
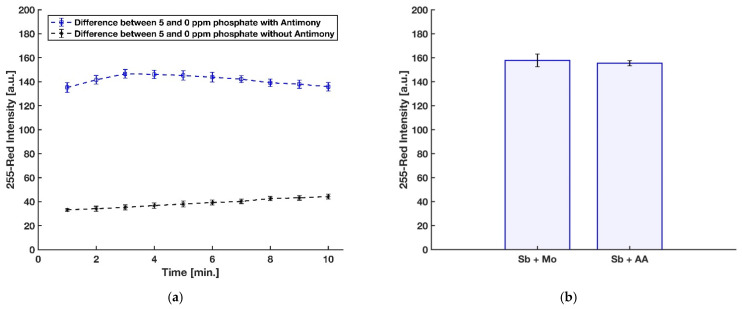
(**a**) Effect of having antimony with ascorbic acid on color intensity. (**b**) Effect of dissolving antimony with molybdenum reagent or with ascorbic acid reagent (*n* = 3 and the error bars show the standard deviation).

In a recent investigation by Divya et al. [45], in contrast with other reports, a negative effect of using antimony on the formation of molybdenum blue complex and its sensitivity for detection of phosphate was recorded. However, their 3 mL final reaction mixture was being heated for incubation at 100 °C for 20 min in order to further enhance the rate of the reduction. However, the heating process can cause some problems, including increasing the effect of silicon interference [48] as well as degrading the final molybdenum blue product [49]. To this end, the impact of Sb in the detection of phosphate for the present device was evaluated with three replications by comparing the red intensity obtained from the detection zone of the device with antimony dried on blotting paper along with ascorbic acid and the device without antimony on the reduction reagent dried on the detection zone. As shown in Figure 7a, Sb is needed to have a rapid formation with an intense color of the molybdenum blue on the detection zone. In addition, in the previously reported paper-based devices for phosphate detection [29,30,31,32], antimony was added to the acidic molybdenum reagent. Therefore, the effect of dissolving antimony with molybdate in DI water or mixing with ascorbic acid and using it as part of the reducing reagent was studied by comparing the blue color product formed on the detection zone with these two approaches, and the result can be seen in Figure 7b. For further study, a two-tailed t-test at 95% confidence level was applied to statistically compare the results obtained by the two procedures. Considering the critical t-value of 2.776 and the calculated experimental t-value of 0.707, no significant difference in the precision of the two methods was observed (*p*-value = 0.519, *df* = 4). However, a liquid reagent consisting of dissolved Sb with molybdate in DI water is not stable and is turned to turbid, presumably because of hydroxide salts [24].

### 3.4. Calibration Curve and Detection Limit in DI Water

**Figure 8:**
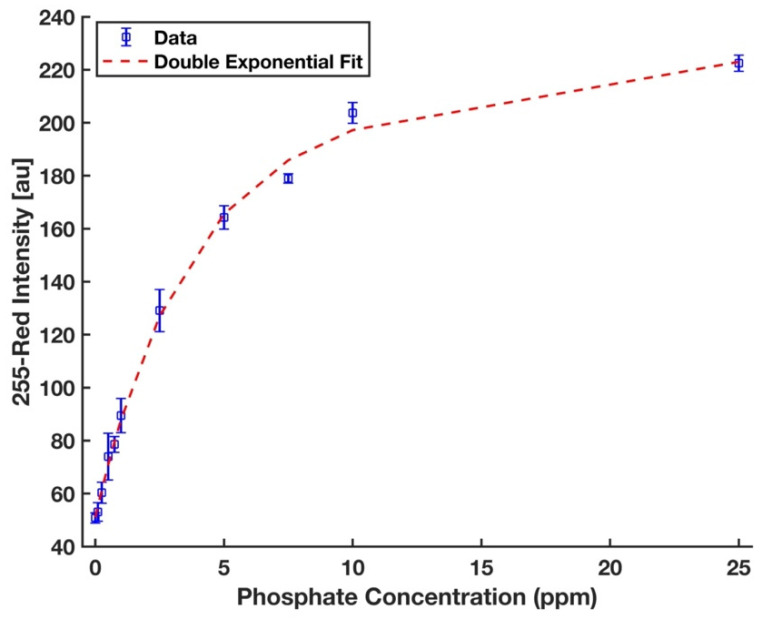
A double exponential calibration curve for phosphate solutions in the form *y* = *a*_1_ × exp *(b*_1_ *× x)* + *a*_2_ *× exp (b*_2_ *× x)*, where *a*_1_ = 194.4, *b*_1_ = 0.006, *a*_2_ = −143.3, *b*_2_ = −0.286, and *R*^2^ = 0.997. The error bars represent the standard deviation of three measurements.

A completely randomized test order with three replicates was run for each concentration of phosphate sample. MATLAB curve fitting toolbox was utilized to fit a double exponential curve of the form *y* = *a*_1_ × *exp* (*b*_1_ × *x*) + *a*_2_ × *exp* (*b*_1_ × *x*) since a nonlinear regression has been often used to fit a model to the entire range of analytical data in the literature [31,50,51]. The symbolic math toolbox in MATLAB was then used to solve the Equations (3) and (4) to calculate the limits of detection (LOD) and quantification (LOQ), respectively [52],

(3) $$y_{LOD}=y_{blank}+3\sigma_{blank}$$

(4) $$y_{LOQ}=y_{blank}+10\sigma_{blank}$$

where *y_blank_* is the mean intensity of the blank (0 ppm); *σ_blank_* is its standard deviation. Figure 8 shows that the calibration curve developed for the device and the chemistry protocol proposed in this work to detect phosphate in deionized water was fitted well to the entire range of data (*R*^2^ = 0.997). The limits of detection and quantification are 0.134 ppm and 0.472 ppm, respectively. For evaluation of the reproducibility of the device performance, 5 ppm phosphate was studied with five replicates, and the percent of relative standard deviation (%RSD) value was 1.8%, which indicates a highly reproducible performance.

### 3.5. Calibration Curve and Detection Limit in Seawater with Visible and Infrared Illumination

**Figure 9:**
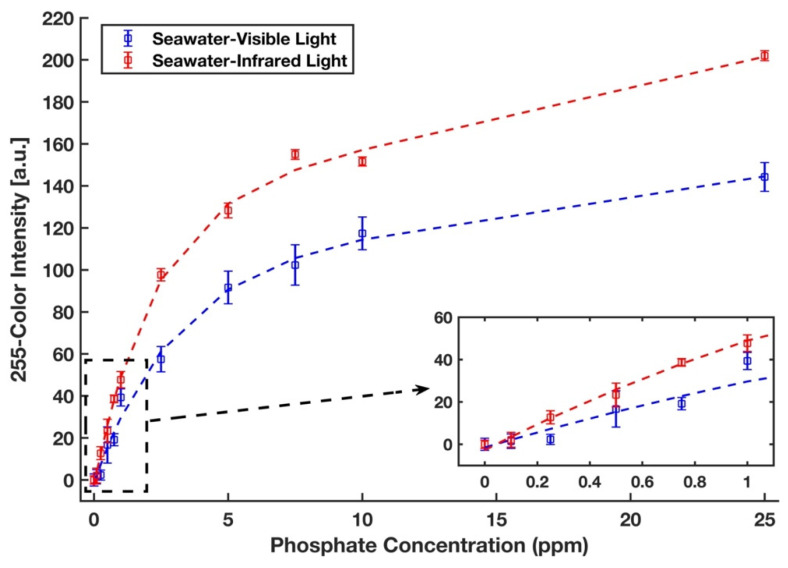
Calibration curves for phosphate in the Sargasso Sea seawater. Signals were taken from the scanner with the visible light (blue) and from the lightbox with the infrared light (red). The error bars represent the standard deviation of three measurements.

The phosphate detection efficacy of the device was also examined in a real seawater sample from the Sargasso Sea, which differs in both salinity and traces of ion content from DI water. Therefore, a completely randomized test order with three replicates was run for each concentration of phosphate sample in the Sargasso Sea seawater. The images of the detection zones were captured in the visible light conditions by the scanner and in the near-infrared zone by a recently developed infrared lightbox that is wirelessly controlled by a smartphone and communicates with it [39]. The red intensity color was selected for the analysis of the images taken by the scanner. The unweighted grayscale intensity was used to create the calibration curve for the images captured by the lightbox. Figure 9 represents the calibration curves with an inset plot for lower phosphate concentrations for the detection of phosphate in Sargasso Sea seawater in visible (blue) and infrared (red) illuminations in which all values were subtracted by a corresponding blank value. Calibration curves in the form *y* = *a*_1_ × *exp* (*b*_1_ × *x*) + *a*_2_ × *exp* (*b*_1_ × *x*) were generated, where *a*_1_ = 59.78, *b*_1_ = −0.345, *a*_2_ = 127.7, *b*_2_= −0.024, and *R*^2^ = 0.992 for the visible light and *a*_1_ = 51.83, *b*_1_ = −0.525, *a*_2_ = 83.33, *b*_2_ = −0.009, and *R*^2^ = 0.995 for the infrared light. The limits of detection and quantification for phosphate with visible illumination are 0.438 ppm and 1.961 ppm, respectively. By using the portable infrared lightbox, the device would not only be a fully portable and field-deployable one, but it can also detect phosphate in the lower concentrations as limits of detection and quantification in the infrared zone are 0.156 ppm and 0.769 ppm, respectively. This accounts for an enhancement in sensitivity by a factor of three and is due to the fact that the maximum absorption of the molybdenum blue occurs in the infrared region of the spectrum (around 850 nm) [53].

### 3.6. Stability of the Device

The main deficiencies of the previously reported paper-based devices for the detection of phosphate in water were the stability of reagents and the lifetime of the devices. The acidic molybdenum reagent in which molybdate dissolves in sulfuric acid tends to reduce after a while due to the auto-reduction of Mo (VI) to Mo (V) [21,26]. In contrast, molybdate dissolved in DI water by itself has a long shelf life, and the same is true for sulfuric acid. Having antimony with molybdate at an aqueous form reduces the stability of the molybdenum reagent since antimony slowly leans towards precipitation. In the present method, antimony was added to ascorbic acid and dried on the detection zone. It has been demonstrated that in the molybdenum blue method in phosphate detection, acidified molybdate does not interact with antimony and, more importantly, antimony does not change the intervalence charge-transfer (IVCT) bands of the pre-formed phosphomolybdenum blue [18,21]. Our results also showed that there are no discernable differences in the color intensity of the complex formed in the detection zone when antimony is present in the molybdate reagent or in the reducing agent. The ascorbic acid reagent in liquid form is also not stable due to the formation of dehydroascorbic acid [29], while according to the supplier, it is stable in powder form for several years at room temperature if stored in dark and dry conditions. Our experiments also show that ascorbic acid is stable for several months in dried form on blotting paper, which is made up of pure cellulose fibers without any additives. To this end, the stability assessment of the device was performed by comparison between the color developed on the detection area of new devices and the device stored under ambient conditions for four months with 5 ppm phosphate samples. The experimental t-value between two devices was 0.43, considering the critical two-tailed t-value of 2.31 for a degree of freedom of 8 at the 95% confidence level (*p*-value = 0.68), no statistically significant difference between results obtained by freshly fabricated devices and those stored under ambient conditions after four months was observed (*M*_1_ = 159.7, *SD*_1_ = 2.1; *M*_2_ = 158.8, *SD*_2_ = 4.3).

The lifetime of this device depends on the stability of its different components, mainly the chemical reagents being used. The molybdenum reagent is indefinitely stable in room conditions [36,37]. The sulfuric acid is stable for two years, and the ascorbic acid did not show any signs of degradation in color (turning into yellow) or performance (experimental two-tailed t-test) after four months. Therefore, if the test strips are stored in an ultraviolet-resistant container with moisture absorbent, it is expected that sulfuric acid will be the limiting factor in the shelf life of this device, i.e., two years.

### 3.7. Comparison of Results

Jayawardane et al. [25] described a 3D colorimetric paper-based device in which molybdate/antimony dissolved in sulfuric acid and ascorbic acid reagent combined together after applying a liquid sample onto the paper. While their device could detect phosphate in the parts per billion (ppb) range, it suffers from a short shelf life and the requirement for storage under frozen conditions. Ribeiro [30] and Racicot et al. [31] tried to solve this problem by adding ethylene glycol as a stabilizer for the acidic molybdate/antimony reagent and reported a 2D colorimetric paper-based device. However, our tests have demonstrated that using ethylene glycol not only reduces the sensitivity of the device but also significantly increases the viscosity of the reagent, making the flow extremely slow in microfluidic channels as well. Moreover, operating this 2D device is a time-consuming procedure that is not in line with the aim of paper-based devices for rapid and point-of-site testing. This is the case since in the device they reported in [30,31], the ascorbic acid reagent needs to be added to the device in four separate 3 μL aliquots and requires a waiting time of at least 20 min between each ascorbic acid addition prior to use of the device. Additionally, the sequence of reaction is not ideal since the reagents mix with each other before sample addition, which results in unwanted reagent reduction. Some commercial paper-based test strips with a long shelf life are also available in the market for detecting phosphate in water. However, their main drawback is their low sensitivity based on the qualitative results obtained via a color chart [39]. Recently, electrochemical paper-based devices (ePADs) have received extensive attention due to their advantages, such as eliminating subjective color comparison by users, high stability, and high selectivity [54]. Cinti et al. [55] developed an ePAD with screen-printed electrodes and wax-printed reaction zones to detect phosphate in water based on the voltammetric measurement of the formation of the phosphomolybdic complex. In order to collect and analyze data, as well as make their device suitable for onsite operation, a portable electrochemical instrument in connection with a laptop was utilized. While this electrochemical sensor could detect a low concentration of phosphate in a few minutes, the preparation procedure was relatively complicated. By using the dip strip developed in this paper, an individual operator can easily detect phosphate in the parts per billion range in the field. Ammonium molybdate dissolved in DI water and the sulfuric acid are separately stored in plastic and glass dropper bottles, respectively, and thus remain stable for two years under room conditions. For further assessment, a comparison is provided in Table 1 between the testing conditions and results attained by the paper-based dip strip presented in this work and those obtained with paper-based devices previously reported.

## 4. Conclusions

In this paper, a new dip strip paper-based device that uses a wet chemistry approach was reported to detect phosphate in water samples, including real seawater. This device overcomes the drawbacks observed in previous paper-based devices. The main advantages of the device developed in this paper is an increased shelf life, improved reproducibility of results, simpler design, and decreased testing duration. The limits of detection and quantification for this device are 0.134 ppm and 0.472 ppm for DI water and 0.438 ppm and 1.961 ppm for the Sargasso Sea seawater, respectively. This device is also fully compatible to integrate with a portable imaging lightbox for on-site phosphate measurements with the limits of detection and quantification as low as 0.156 ppm and 0.769 ppm, respectively. Future work will include implementing paper-based actuators to adapt this wet chemistry approach into an autonomous paper-based platform [56]. Additionally, further research using this wet chemistry protocol could be done to detect phosphate in other matrices such as food, soil, and saliva samples.

## Figures and Tables

**Table 1:** Comparison of detecting phosphate in DI water using the dip strip in this study with previously reported devices.

Device	Working Range (ppm)	LOD (ppm)	Repeatability	Reaction Time (min.)	Shelf Life	Ref.
3D colorimetric paper-based device	0.6–30	0.153	Less than 2% RSD	40	122 days stored in freezer at <−20 °C	[29]
2D colorimetric paper-based device	0.1–10	0.160	N/A	4	9 months in refrigerator at <4 °C	[31]
Quantofix phosphate test kit	0.1–50	1.352	2.1% RSD	1	2 years under room temperature	[39]
Electrochemical paper-based device	1–30	0.38	Less than 6% RSD	2.5	30 days at room temperature	[55]
Dip strip with wet chemistry	0.1–25	0.134	1.8% RSD	3	4 months and expected to be 2 years under room temperature	This work

## Data Availability

Data is contained within the article. Additional data not presented in this article is available on request from the corresponding author.
